# Regulation of GABA Equilibrium Potential by mGluRs in Rat Hippocampal CA1 Neurons

**DOI:** 10.1371/journal.pone.0138215

**Published:** 2015-09-21

**Authors:** Bo Yang, Padmesh S. Rajput, Ujendra Kumar, Bhagavatula R. Sastry

**Affiliations:** 1 Neuroscience Research Laboratory, Department of Anesthesiology, Pharmacology & Therapeutics, Faculty of Medicine, The University of British Columbia, Vancouver, Canada; 2 Faculty of Pharmaceutical Sciences, The University of British Columbia, Vancouver, Canada; University of Exeter, UNITED KINGDOM

## Abstract

The equilibrium potential for GABA-A receptor mediated currents (E_GABA_) in neonatal central neurons is set at a relatively depolarized level, which is suggested to be caused by a low expression of K^+^/Cl^-^ co-transporter (KCC2) but a relatively high expression of Na^+^-K^+^-Cl^-^ cotransporter (NKCC1). Theta-burst stimulation (TBS) in stratum radiatum induces a negative shift in E_GABA_ in juvenile hippocampal CA1 pyramidal neurons. In the current study, the effects of TBS on E_GABA_ in neonatal and juvenile hippocampal CA1 neurons and the underlying mechanisms were examined. Metabotropic glutamate receptors (mGluRs) are suggested to modulate KCC2 and NKCC1 levels in cortical neurons. Therefore, the involvement of mGluRs in the regulation of KCC2 or NKCC1 activity, and thus E_GABA,_ following TBS was also investigated. Whole-cell patch recordings were made from Wistar rat hippocampal CA1 pyramidal neurons, in a slice preparation. In neonates, TBS induces a positive shift in E_GABA,_ which was prevented by NKCC1 antisense but not NKCC1 sense mRNA. (RS)-a-Methyl-4-carboxyphenylglycine (MCPG), a group I and II mGluR antagonist, blocked TBS-induced shifts in both juvenile and neonatal hippocampal neurons. While blockade of mGluR1 or mGluR5 alone could interfere with TBS-induced shifts in E_GABA_ in neonates, only a combined blockade could do the same in juveniles. These results indicate that TBS induces a negative shift in E_GABA_ in juvenile hippocampal neurons but a positive shift in neonatal hippocampal neurons via corresponding changes in KCC2 and NKCC1 expressions, respectively. mGluR activation seems to be necessary for both shifts to occur while the specific receptor subtype involved seems to vary.

## Introduction

γ-aminobutyric acid (GABA) plays an important role in regulating neuronal excitability in mammalian central nervous system (CNS). Since the amplitude and direction of GABA_A_ mediated postsynaptic currents (PSCs) are subject to changes in their equilibrium potential (E_GABA_), either activity-mediated shifts [1,2,3] or age-related changes [1,4,5,6] in E_GABA_ can affect GABAergic synaptic transmission, the excitability of target neurons, excitatory synaptic plasticity, and thus, the balance between excitation and inhibition in the CNS.

Since E_GABA_ is mainly determined by the intracellular Cl^-^ concentration ([Cl^-^]_i_) in central neurons [3,7], changes in the activity of K^+^-Cl^-^ cotransporter (KCC2) and Na^+^-K^+^-Cl^-^ cotransporter (NKCC1) can contribute to activity-mediated or age-dependent changes in E_GABA_. Our previous study suggests that, in juvenile rat hippocampal CA1 neurons, shifts in E_GABA_ caused by low-frequency stimulations or theta-burst stimulation (TBS) were due to the upregulation of KCC2 but not NKCC1 activity [3]. Whether there are other factors that contribute to this process is unclear.

Metabotropic glutamate receptors (mGluRs) are involved in various cellular actions including synaptic plasticity in the CNS [8,9]. Based on the homology of amino acid sequences or subunit composition of molecular cloning, mGluRs can be divided into three groups (group I, II and III mGluRs) or mGlu1-8 receptors [10]. In contrast to group II and III mGluRs acting as presynaptic autoreceptors in the hippocampus, the group I mGluRs are mainly expressed on postsynaptic membranes [10,11,12]. Activation of group I mGluRs typically stimulates the mobilization of Ca^2+^ through IP_3_-sensitive stores [13,14] and/or triggers PKC-dependent signalling pathways [15].

KCC2 is expressed in the proximity of excitatory synapses in the hippocampus [16]. Activation of mGluRs (especially group I mGluRs) is involved in an upregulation of KCC2 [15] in cortical neurons. Activation of group I mGluRs leads to an increase in [Ca^2+^]_i_ via voltage-gated Ca^2+^ channels (VGCC) [17]. Therefore, the involvement of mGluRs in the modulation of KCC2 activity and E_GABA_ following TBS, in hippocampal neurons, was examined in this study. Despite of the important role of NKCC1 in the accumulation of Cl^-^ in neurons [18,19], little is known about the modulation of this cotransporter in central neurons. Recent studies suggest that activation of ionotropic glutamate receptors and/or group I mGluRs stimulate NKCC1 activity in cortical neurons [20,21]. Therefore, the involvement of mGluRs in modulating NKCC1 activity following TBS in hippocampal neurons was examined as well in the current study.

One particularly intriguing aspect of E_GABA_ is that it is set at a more depolarized level in neonatal neurons than adult neurons [5,6,22,23], which seems to be related to differences in the expression of KCC2 and NKCC1 during development and maturation of central neurons [24,25]. To our knowledge, no data on the possible involvement of mGluRs in the regulation of KCC2 or NKCC1 in neonatal hippocampal neurons have been presented in the literature.

In our present study, we have addressed the following questions: (1) whether E_GABA_ is shifted following TBS in neonatal hippocampal CA1 neurons and, if any, whether KCC2 or NKCC1 is responsible for this shift; (2) are mGluRs involved in TBS-induced changes in KCC2 or NKCC1 activity and E_GABA_ in both juvenile and neonatal hippocampal neurons; (3) which mGluR subtype is involved in regulation of KCC2 and/or NKCC1 activity following TBS in rat hippocampal neurons?

## Materials and Methods

### Hippocampal slice preparation

Hippocampal acute slices were prepared from postnatal day 9–12 or 3–5 day old male Wistar rats (Animal Care Centre, The University of British Columbia) following a protocol approved by Canadian Council on Animal Care, and maintained as previously described [2]. Animals were euthanized under 4–5% isoflurane deep anesthesia. Briefly, under 4–5% isoflurane induced inhalational anesthesia, the brain was removed from the rat and transverse sections of the hippocampus (400 μm) were cut by using a vibratome (Leica VT1200, Wetzlar, Germany) and CA3 region was removed from the slice to diminish the influence of spontaneous activity in CA3 neurons. Slices were then allowed to recover for 1 h in an incubating chamber, containing (in mM): 120 NaCl, 3 KCl, 1.8 NaH_2_PO_4_, 26 NaHCO_3_, 2 CaCl_2_, 2 MgCl_2_ and 10 dextrose, equilibrated with 95% O_2_-5% CO_2_ carbogen (pH 7.35–7.4). Following the incubation period, slices were transferred into the recording chamber and superfused with the same solution at 1.5–2 ml/min at 25–26°C.

### Electrophysiological recordings

Whole-cell patch clamping was used to record responses from individual hippocampal CA1 pyramidal neurons identified under an upright microscope (Zeiss Axioskop 4 plus, Munich, Germany). Patch pipettes were pulled from thin walled, 1 mm outer diameter, borosilicate glass capillary tubing with a filament (WPI, Sarasota, FL) and the resistance of the final electrode ranged between 5 and 10 MΩ. Recording pipettes were filled with (in mM): 135 K-gluconate, 10 HEPES, 10 KCl, 1 BAPTA, 5 Mg-ATP, 0.1 CaCl_2_, 10 Na_2_-phosphocreatine, 0.4 Na_3_-GTP and creatine phosphokinase 50 U/ml (pH 7.20–7.30). Pipette capacitance was minimized and series resistance (typically in the range of 15–25 MΩ) was monitored throughout the experiments. Recordings were terminated and data discarded if the series resistance changed by more than 15% during a recording. The average resting membrane potential for the recorded CA1 neurons was -65 mV (n = 93) and the average input resistance was 38 MΩ (n = 93).

Perforated patch clamp technique [26,27] was also used in one series of experiment in the current study. Electrodes (glass capillaries) with a resistance of ~5 MΩ were tip filled with intracellular solution (ICS) and then backfilled with the same pipette solution used in whole cell patch clamp recordings but containing 50 μg/ml gramicidin D (diluted from a stock solution of 50 mg/ml in DMSO). The extracellular bath solution and other procedures are the same as used in whole cell patch clamp recordings.

Dl-2-Amino-5-phosphonovaleric acid (APV; 50 μM) and 6,7-Dinitroquinoxaline-2,3-dione (DNQX; 20 μM) were present throughout the recording to block glutamatergic postsynaptic currents (PSCs). Theta-bursts were given in the stratum radiatum as previously described [2,3,28]. PSCs from CA1 pyramidal neurons were evoked at 0.05 Hz control frequency by stimulations in the stratum radiatum as previously described [3]. The control frequency of stimulation (0.05 Hz) was maintained throughout whether the voltage steps are given for assessing E_GABA_ or for stimulating while clamping the cell at -60 mV. The TBS protocol involves giving 4 pulses at 100 Hz in each burst in a train consisting of 5 such bursts with an inter-burst interval of 200 ms; the train is repeated thrice at 30 s intervals. TBS was applied while holding the CA1 neuron under current clamp. The influence of TBS conditioning on E_GABA_, as well as the amplitude and conductance of the PSC were examined every 5 min and data for 30 min post-TBS values are shown as in our previous publications [3]. PSC amplitude was calculated by measuring the peak amplitude and E_GABA_ was then extrapolated from a linear regression of the corrected amplitude vs. holding potential. Corrected amplitude is achieved by subtracting the steady-state current from peak PSC. Conductance value was calculated as the slope of I-V plots. We used platinum bipolar stimulation electrodes placed in the stratum radiatum and a constant current stimulation source to evoke the PSCs in hippocampal CA1 neurons. If the amplitude of the PSC starts to change (increase or decrease) within 20 min after break-in, the cell was discarded.

**Fig 1:**
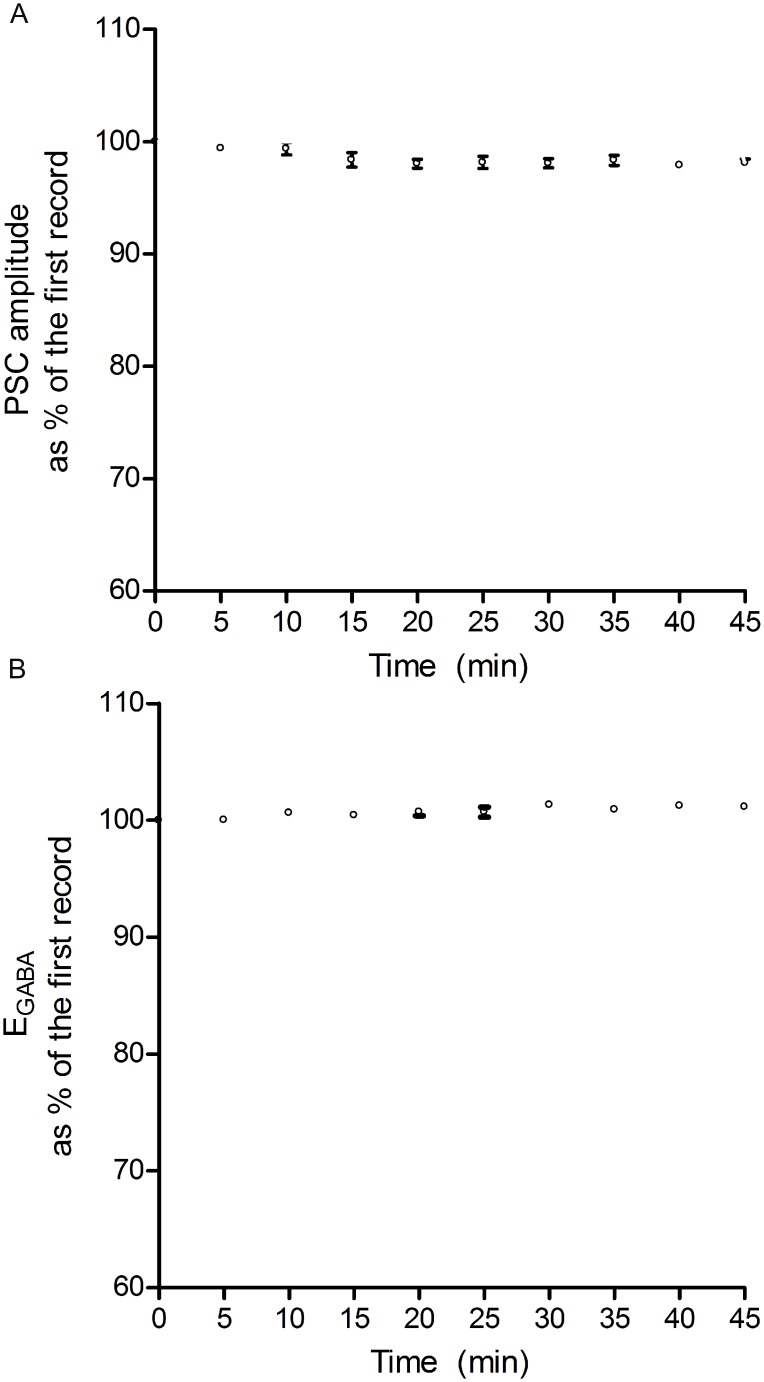
Stability of PSC amplitude and E_GABA_ during control stimulation frequency (0.05 Hz). When evoked at a low frequency (0.05 Hz), PSC amplitude and E_GABA_ were both stable. Note that the values on the Y-axis were normalized as a percentage of control (the first record). Any deviation from the respective controls was small (less than 5%) and not statistically significant and, hence, used subsequently as a control stimulation frequency throughout the study. Data in panel A (PSC amplitude) and B (E_GABA_) were obtained from hippocampal CA1 neurons (n = 6) in 9–12 day old rats. Individual points on panel A and the corresponding points on B are from the same neurons. All recordings were made using the whole-cell recording method. The amplitudes plotted in panel A were taken while clamping the neurons at- 60mV.

In our experiments, after getting the whole-cell configuration, control responses were recorded for 10 min following a 10–20 min initial stabilization period. When recorded in this fashion, we did not observe any significant change to E_GABA_ in control responses for about 1 hour [2,3,28]. Perforated patch recordings is generally thought to clamp the intracellular Cl^-^ gradient and yield a more accurate calculation of E_GABA_, however, this technique does not allow the intracellular application of drugs or agents (such as NKCC1 sense or antisense mRNA, used in the current study). Since E_GABA_ and the amplitude of GABA-ergic IPSC are stable when the IPSC is evoked at 0.05 Hz using the whole-cell patch clamp recording method ([3]; Fig 1), it is employed in the current study.

NKCC1 antisense and sense mRNA were loaded intracellularly as described previously [3,28]. The recording was initiated 15 min after the whole-cell configuration so that antisense or sense mRNAs could equilibrate with cell content. Even though the exact turnover rate of NKCC1 mRNA is unknown, while E_GABA_ was monitored every 5 min, 30 min post-TBS was taken as a time point to quantitatively express possible changes in E_GABA,_ as described previously [3].

### Western blot analysis

To determine the expression of KCC2 and NKCC1, western blot analysis was performed in tissue lysate prepared from 3–5 and 9–12 day old rats hippocampus as described previously [29]. Briefly, hippocampus tissue slices from control, TBS conditioning and mGluR antagonist (MCPG) treated were homogenized in homogenizing buffer containing (62.5mM Tris-HCl, 50mM dithiothretiol [DTT], 10% glycerol, 2% SDS); 20μg of total protein prepared in Laemmli sample buffer was subjected to on 7% SDS-PAGE and transferred to nitrocellulose membrane in transfer buffer (20mM Tris, 192mM glycine and 20% methanol). The membrane was blocked with 5% non-fat dried skim milk at room temperature for 1 h and incubated overnight at 4°C in presence of specific primary antibodies for KCC2 (1:1000) and NKCC1 (1:1000) diluted in 5% bovine serum albumin. The membrane was later incubated with peroxidase conjugated goat anti-rabbit secondary antibody at room temperature for 1 h. The bands were detected using the chemiluminescence detection system and photographed on Alpha Innotech FluorChem 8800 (Alpha Innotech Co., San Leandro, CA) gel box imager. β-actin was used as the housekeeping protein for loading control. The bands were quantified using densitometric analysis and protein expression was calculated as the ratio of band of interest and the density of β-actin.

### Chemicals

Dl-APV, DNQX, (2S)-a-Ethylglutamic acid (EGLU) and (S)-(+)-a-Amino-4-carboxy-2-methylbenzeneacetic acid (mGluR1 antagonist, LY367385) were purchased from Tocris (Ellisville, MO) and MCPG (group I and II mGluR antagonist), 2-Methyl-6-(phenylethylnyl)pyridine hydrochloride (MPEP, mGluR5 antagonist) were purchased from Ascent Scientific (Bristol, UK). The primary anti-rabbit KCC2 antibody and gramicidin D were purchased from Sigma (St. Lois, MO) and NKCC1 antibody was provided by ProteinTech Group Inc. (Chicago, IL). Goat anti-rabbit Alexa-594 secondary antibody was purchased from Invitrogen (Burlington, ON, Canada). NKCC1 sense and antisense mRNAs were custom ordered from Invitrogen Life Technologies (Burlington, Ontario, Canada). The sequences for oligodeoxynucleotides (oligos) are as follows: NKCC1 sense--5'-GTCATCACAAGAAAAGTCACCTGGTACCAAGGATGT-3'); NKCC1 antisense--5'-ACATCCTTGGTACCAGGTGACTTTTCTTGTGATGAC-3'). Sense and antisense oligodeoxynucleotides were dissolved in the recording solution from concentrated stock solutions and the final concentration of sense or antisense mRNA in the pipette was 100 nM.

### Data acquisition and analysis

PSCs were recorded in the whole-cell voltage clamp mode using an Axopatch 200A (Molecular Devices, Sunnyvale, CA) amplifier. Records were digitized using Digidata 1322A interface and Clampex Ver. 9.0 software (Molecular Devices, Sunnyvale, CA). Data were acquired at 5 kHz and low pass filtering was set at 2 kHz. Electrophysiological data were analyzed using Clampfit Ver. 9.2 software (Molecular Devices, Sunnyvale, CA) and expressed as mean ± SEM. Statistical analysis of most electrophysiological data was performed using a two-tailed paired Student’s t-test. One way ANOVA analyses were used in two series of electrophysiological study and two western blot experiments involving multiple group comparisons. The level of significance (p value) was arbitrarily chosen to be <0.05 for both electrophysiological data and western blot analysis. Since one cell per hippocampal slice was used in these studies, *n* refers to number of cells. A total of 93 slices from 62 animals were used for this study.

## Results

### Stability of the PSC and E_GABA_

When the IPSC is evoked at 0.05 Hz, its amplitude as well as E_GABA_ were stable during a 45 min recording period (n = 6; Fig 1); the records taken at 5–45 min were not significantly different from the first one (0 time). These results are consistent with our previous studies [2,3]. Therefore, 0.05 Hz was chosen as the control stimulation frequency throughout this study.

### Comparison of perforated-patch recording with whole cell patch clamp recording

**Fig 2:**
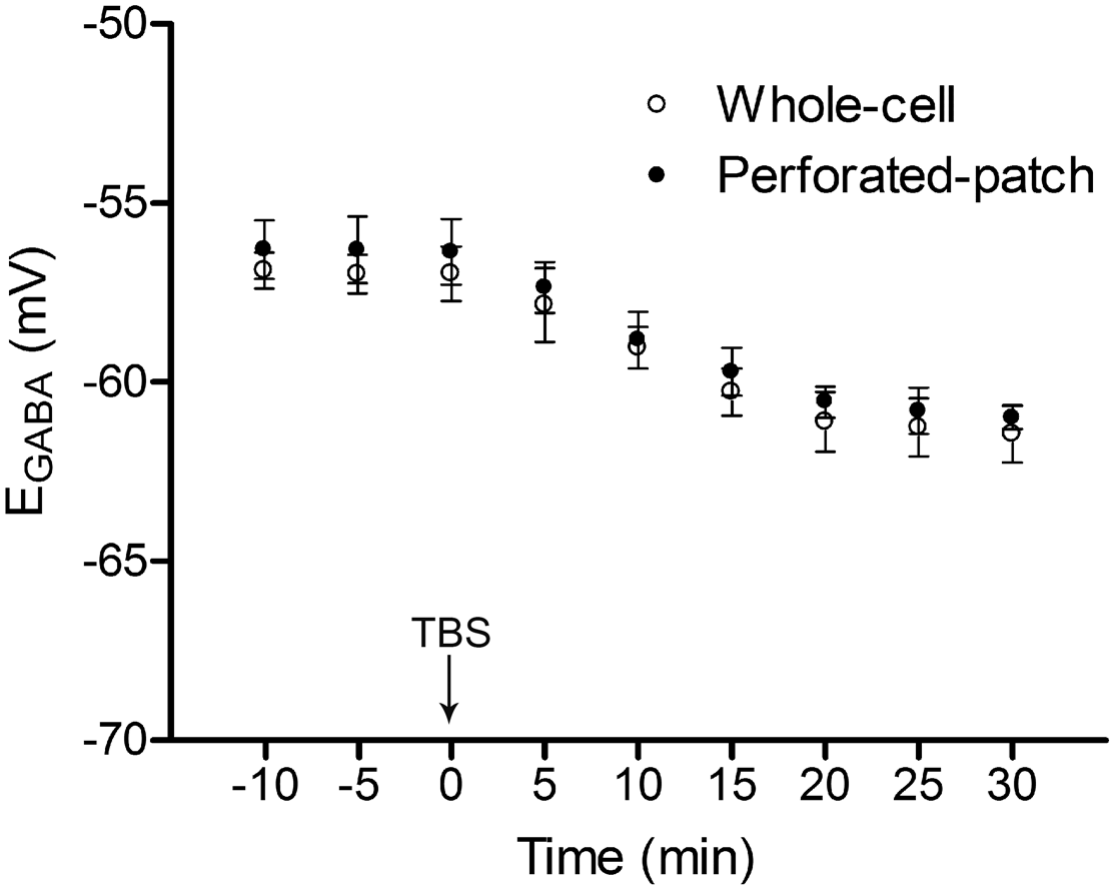
Comparison of TBS-induced changes in E_GABA_ using whole-cell and perforated-patch recording methods. Traditional whole-cell patch clamping and perforated-patch recording were compared in two series of experiments. All the recordings were monitored for 10 min under 0.05 Hz stimulation before the TBS was delivered (at 0 min on X-axis). Note that shifts in E_GABA_ occur in hippocampal CA1 neurons of 9–12 day old rats following TBS in both methods (n = 6 for each). There was no statistical difference between records using the two methods. E_GABA_ was calculated using the method described in “Materials and Methods” section.

Our previous studies suggest that TBS in the stratum radiatum induced a negative shift in the E_GABA_ in juvenile rat hippocampal CA1 neurons [2]. In order to further examine whether the shift in E_GABA_ observed following TBS in hippocampal neurons was just an “artifact” brought by whole cell patch recording, gramicidin perforated patch clamp was used as well in one series of experiments. In gramicidin D (50 μg/ml) loaded hippocampal CA1 neurons, following TBS of the input, E_GABA_ was shifted in the negative direction (E_GABA pre-TBS_: -56.3±1.1, E_GABA-30min post-TBS:_ -61.0±0.8 mV, n = 6, p<0.05) while PSC conductance was not significantly changed (before TBS: 5.9±0.3 nS, 30 min after TBS: 5.5±0.4 nS, n = 6, P>0.05). In addition, E_GABA_ was not changed in the presence of gramicidin D during the observation period (0 min: -56.1±0.8, 30 min: -56.6±1.0 mV, n = 6; p>0.05) in another set of experiment (without TBS conditioning), suggesting that shifts in E_GABA_ were not caused by gramicidin but TBS conditioning. These data suggest that the shift in E_GABA_ also occurs following TBS in hippocampal CA1 neurons under perforated patch configuration. We compared the perforated-patch method with the whole cell-patch method while recording the IPSCs and measuring E_GABA_; there were no significant differences with using either method (Fig 2). Perforated-patch method requires a lot of time to stabilize and also is not conducive to injecting sense and antisense agents into cells (used in some of the experiments). Since the studies involve plasticity requiring a healthy slice preparation, we do not use slices 2 hours past their preparation, making the perforated patch method unproductive. Therefore, in subsequent sets of experiments, the whole cell-patch recording method was employed.

### TBS induces a positive shift in E_GABA_ in neonatal rat hippocampal neurons

**Fig 3:**
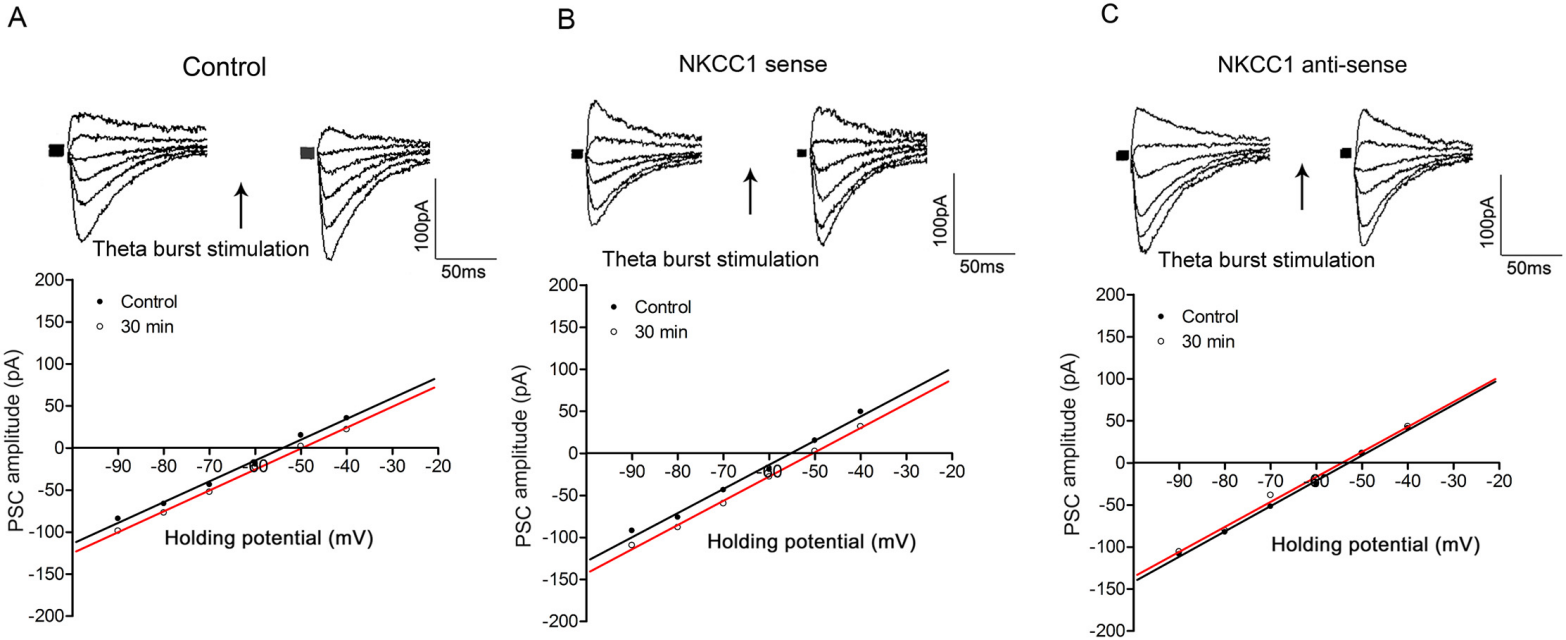
NKCC1 antisense blocks TBS-induced positive shift in E_GABA_ in neonatal neurons. In panel A, TBS-induced positive shift in E_GABA_ was observed 30 min following TBS in control cells. The positive shift was still observed in cells loaded with NKCC1 sense mRNA (panel B), while completely blocked in NKCC1 antisense mRNA loaded cells (panel C). Note the PSC conductance was not changed pre- and post TBS in both cases. In each panel, PSC records (on the top) were evoked at different holding potentials and I-V plots (on the bottom) represented corrected PSC amplitudes at various holding potentials. In all panels, the left PSC records are controls and the right ones are taken 30 min following TBS conditioning. Data in panels A, B & C were obtained from three different CA1 neurons from 3–5 day old rats and the holding potential for each individual cell was -60 mV. The whole-cell recording method was used in this study.

Thirty minutes following TBS of the input, E_GABA_ was shifted in a positive direction in neonatal hippocampal CA1 neurons (E_GABA pre-TBS_: -53.5±0.5 mV, E_GABA-30min post-TBS:_ -49.1±1.0 mV, n = 6, P<0.05, Fig 3A), in contrast, with a shift in a negative direction in juvenile neurons as described previously (Yang et al., 2010). In order to further investigate the possible involvement of NKCC1 in this process, we applied an antisense technique as described in our earlier reports [1,3]. Interestingly, in cells loaded with the NKCC1 antisense mRNA, the E_GABA_, was not significantly changed (E_GABA pre-TBS_: -52.9±0.7 mV, E_GABA-30min post-TBS_: -53.9±0.8 mV, n = 6; p>0.05, Fig 3C) and the PSC conductance was also not changed (before TBS: 4.9±0.6 nS, 30 min after TBS: 4.2±0.5 nS, n = 6, P>0.05). However, in cells loaded with NKCC1 sense mRNA, TBS-induced positive shift in E_GABA_ was still observed (E_GABA pre-TBS;_ -54.1±0.5 mV, E_GABA-30min post-TBS_: -49.9±0.4 mV, n = 6; p<0.05, Fig 3B). Sense or anti-sense mRNA did not affect the resting properties of the neurons such as input resistance or membrane potential (sense mRNA loaded neuron: input resistance 36.2±1.0 MΩ, membrane potential -64±1.5 mV, n = 6; antisense mRNA loaded neuron: input resistance 34.6 ±0.8 MΩ, membrane potential -61.5±1.2 mV, n = 6, P>0.05) compared to control neurons (input resistance 34.3±0.8 MΩ, membrane potential -62.5±0.6 mV, n = 6). These data indicate that NKCC1 activity is involved in TBS-induced depolarizing shift in E_GABA_ in neonatal hippocampal neurons.

### mGluRs are involved in TBS-induced shifts in E_GABA_ in both juvenile and neonatal neurons

**Fig 4:**
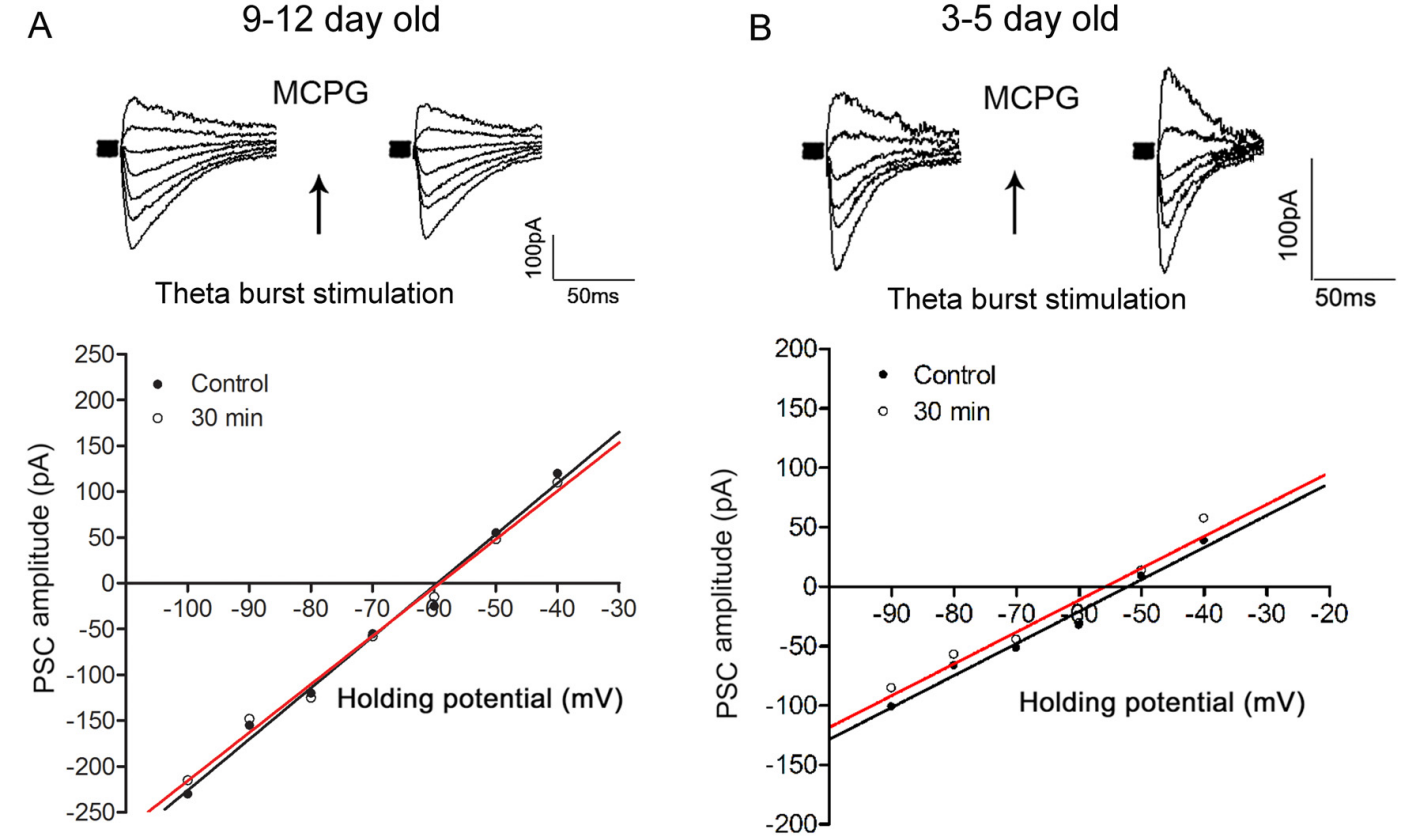
MCPG blocks TBS-induced shifts in E_GABA_ in both juvenile and neonatal rat hippocampal neurons. MCPG (500 μM) was applied into superfusion medium 10 min prior to TBS conditioning. In panels A & B, the effects of MCPG on the TBS-induced shifts in E_GABA_ are shown from 9–12 and 3–5 day old rat hippocampal neurons, respectively. Data were obtained from two different CA1 neurons and holding potential for each individual cell was -60 mV. Note that MCPG blocked TBS-induced negative shift in E_GABA_ in both juvenile and neonatal rat hippocampal neurons. The whole-cell recording method was used.

Results from our previous [3] and present study suggest that an up-regulation of KCC2 activity is associated with TBS-induced negative shift in E_GABA_ in juvenile neurons while an up-regulation of NKCC1 activity contributes to a positive shift in E_GABA_ in neonatal neurons (Fig 3). To examine the involvement of mGluRs in the modulation of KCC2 activity and E_GABA_ following TBS in hippocampal neurons, a group I and II mGluR antagonist, MCPG, was used in this study. MCPG at a concentration at 500 μM (TBS was applied after 10 min of the drug application and drug application continued for another 10 min), blocked the TBS-induced shifts in E_GABA_ in either juvenile or neonatal neurons (in juvenile: E_GABA pre-TBS_: -59.8±2.3 mV, E_GABA-30min post-TBS_: -59.0±2.5 mV, n = 6; p>0.05; in neonate; E_GABA pre-TBS_: -51.7±0.5 mV, E_GABA-30min post-TBS_: -53.3±0.7 mV, n = 6; p>0.05, Fig 4). In addition, E_GABA_ was unaffected by MCPG alone (no TBS) in both juvenile (no drug: -58.6±2.5 mV; in drug: -59.8±2.3 mV, n = 6, P>0.05) and neonatal (no drug: -51.5±1.1 mV; in drug: -51.7±0.5 mV, n = 6, P>0.05) neurons. Taken together, these results suggest that in rat hippocampal CA1 pyramidal neurons, activation of mGluRs is involved in TBS-induced negative shift in E_GABA_ in juveniles or positive shift in E_GABA_ in neonates. Since MCPG can block group I (mGluR1 and mGluR5) and group II receptors (mGluR2 and mGluR3), a decision was made to examine, in the next set of experiments, the actions of group I (MPEP [blocks mGluR5] and LY367385 [blocks mGluR1]) and group II (EGLU) mGluR antagonists, to determine which, if any, of these receptor subtypes is involved.

### Activation of mGluR1 or mGluR5 alone is sufficient to induce a negative shift in E_GABA_ following TBS in juvenile rat hippocampal neurons

**Fig 5:**
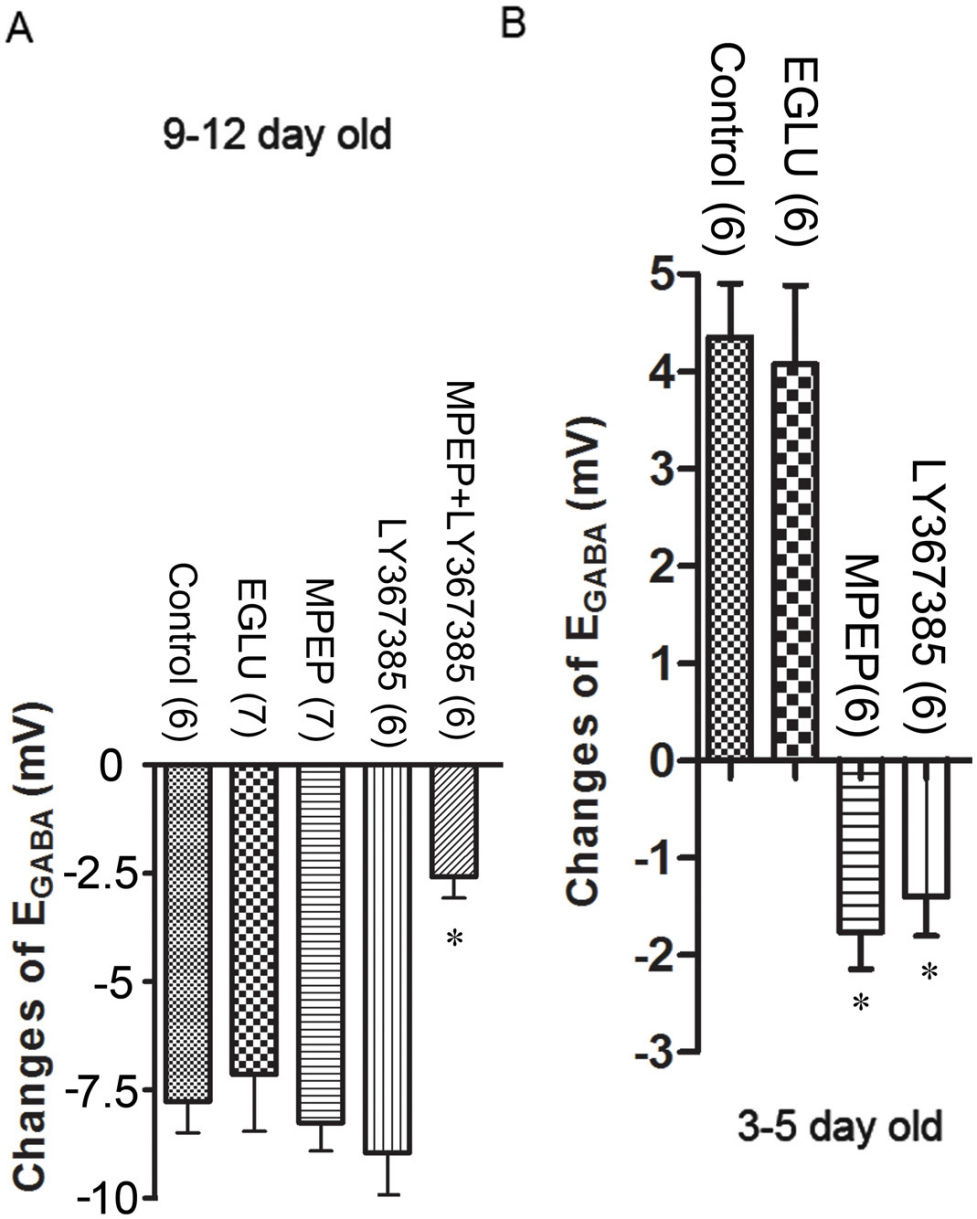
Different effects of mGluR antagonists on TBS-induced shifts in E_GABA_ in juvenile and neonatal rat hippocampal neurons. In panel A, MPEP and LY367385 did not block TBS-induced negative shifts in E_GABA_ as observed in control condition (reported in [2,3] in 9–12 day old rat hippocampal neurons while the co-application of these two antagonists blocked this negative shift in E_GABA_. In panel B, either MPEP or LY367385 is able to inhibit TBS-induced positive shifts in E_GABA_ in 3–5 day old rat hippocampal neurons. EGLU didn’t block the TBS-induced shifts in both 9–12 and 3–5 day old rat hippocampal neurons. The *n* in brackets refers to the number of neurons. *P<0.05.

To test which mGluR subtype is involved in the E_GABA_ shifts, several mGluR antagonists (MPEP, LY367385, EGLU) were examined. Concentrations and durations of applications of these agents that did not have a significant effect on either the IPSC amplitudes or E_GABA_ were first determined for each drug before examining their effects on TBS-induced changes in E_GABA_. A 10 min application of MPEP (10 μM) or LY367385 (100 μM) or EGLU (10 μM) in the superfusing medium and giving TBS conditioning after 5 min in the drug, was chosen for subsequent experiments. EGLU did not block the TBS-induced shift in E_GABA_ (E_GABA pre-TBS_: -57.7±0.6 mV, E_GABA-30min post-TBS_: -61.7±0.7 mV, n = 6; p>0.05 vs. control, Fig 5), suggesting group II mGluRs seem not to be involved in shifts in E_GABA_. When TBS was given in the presence of MPEP or LY367385, the TBS-induced shift in E_GABA_ was still observed in juvenile rat hippocampal neurons (MPEP: E_GABA pre-TBS_: -59.9±1.6 mV, E_GABA-30min post-TBS_: -68.5±1.5 mV, n = 6; p>0.05 vs. control; LY367385: E_GABA pre-TBS_: -59.0±1.5 mV, E_GABA-30min post-TBS_: -66.8±1.1 mV, n = 7; p>0.05 vs. control, Fig 5). However, in a separate set of experiments, when MPEP and LY367385 were co-applied for 10 min and TBS was given after 5 min in drugs, the TBS-induced shift in E_GABA_ in juvenile hippocampal CA1 neurons was significantly reduced (E_GABA pre-TBS_: -58.5±1.0 mV; E_GABA-30min post-TBS_: -61.0±0.9 mV, n = 7; P<0.05, Fig 5) compared to when the drugs were applied individually or no drug was applied, during TBS. The above combined application of MPEP and LY367385 did not have any significant effect on the pre-TBS (control) E_GABA_. Therefore, a blockade of both mGluR1 and mGluR5 is needed to block the induction of TBS-mediated negative shift in E_GABA_ in juvenile neurons.

### Co-activation of mGluR1 and mGluR5 is needed for TBS-induced positive shift in E_GABA_ in neonatal neurons

When EGLU (10 μM) was applied into superfusion medium for 10 min, the TBS-induced shift in E_GABA_ was still observed in neonatal neurons (E_GABA pre-TBS_: -55.0±0.9 mV, E_GABA-30min post-TBS_: -50.9±0.3 mV, n = 6; p>0.05 vs. control, Fig 5), suggesting group II mGluRs seem not to be involved. When neonatal slices were superfused with either MPEP or LY367385 for 10 min and TBS conditioning was given at 5 min of the drug application, there was no positive shift in E_GABA_ (in fact, negative shifts were observed) 30 min following TBS (Fig 5), suggesting that, unlike in juvenile neurons, a co-activation of mGluR1 and mGluR5 is needed to induce a positive shift in E_GABA_ in neonatal neurons.

### Differential expression of KCC2 and NKCC1 in juvenile and neonatal rat hippocampus

Previous studies have shown that expression of KCC2 is relatively higher than that of NKCC1 in juvenile rat hippocampus while expression of NKCC1 is higher than KCC2 in neonatal hippocampus [6,30,31]. Since our studies suggest that TBS induces a negative shift in E_GABA_ in juveniles and a positive shift in neonates, and it is known in literature that KCC2 contributes to a negative shift and NKCC1 contributes to a positive shift in E_GABA_, we decided to determine whether TBS affects the levels of these co-transporters in the hippocampus, using western blot analysis; we also examined the effects of mGluR antagonist MCPG on the expression of these cotransporters.

**Fig 6:**
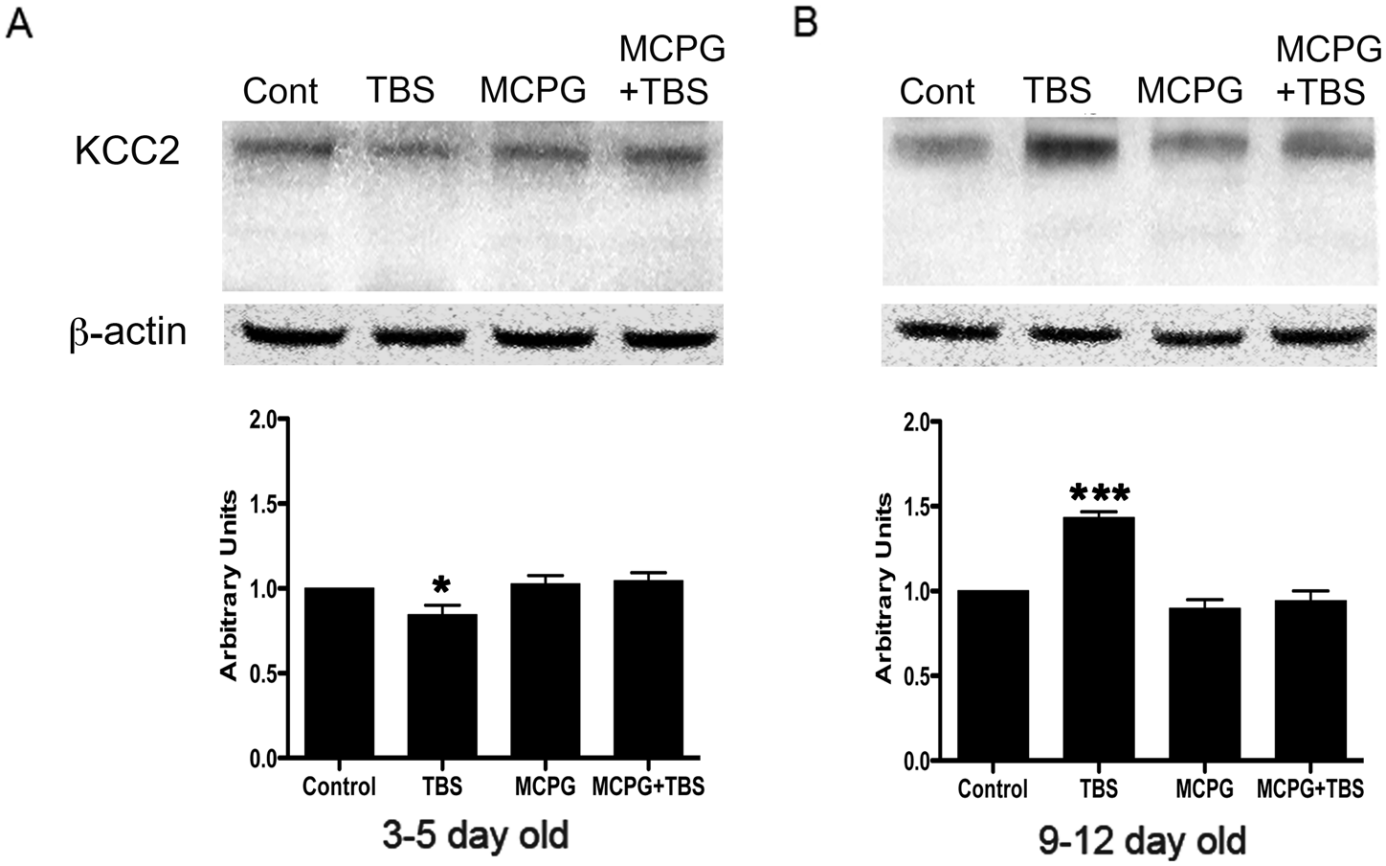
Western blot analysis of KCC2 in 3–5 day and 9–12 day old rat hippocampus. Total tissue lysate (20 μg) from CA1 region of hippocampus was subjected to immunoblot analysis using KCC2 specific antibodies. KCC2 expression was detected at the expected size of 140 kDa. Note the increased expression of KCC2 following TBS in the CA1 area of 9–12 day old rat hippocampus (panel B) while decreased expression of KCC2 in that of 3–5 day old rat hippocampus (panel A). KCC2 expression was not significantly changed in MCPG and MCPG plus TBS treated slices when compared to control in both 3–5 day old (n = 5) and 9–12 day old (n = 5) rat hippocampus. β -actin as loading control and densitometric analysis are shown in middle and bottom panel, respectively. Data are presented as mean ± SD, * P<0.05,***P<0.001.

In western blot, KCC2 was detected at the expected size of 140 kDa (Fig 6). In the 3–5 day old rat, low KCC2 like immunoreactivity was detected in controls and with TBS conditioning, however, the expression of KCC2 was significantly reduced after TBS compared to that in the controls. Although a slightly increased expression was seen upon MCPG or MCPG+TBS treatment, the expression was not significantly different from controls. In the hippocampus of the 9–12 day old rat, KCC2 was well expressed in controls and the expression was significantly increased upon TBS conditioning. KCC2expression following MCPG or MCPG+TBS treatment was comparable to controls (Fig 6), raising the possibility of mGluR involvement in the TBS-induced KCC2 up-regulation.

**Fig 7:**
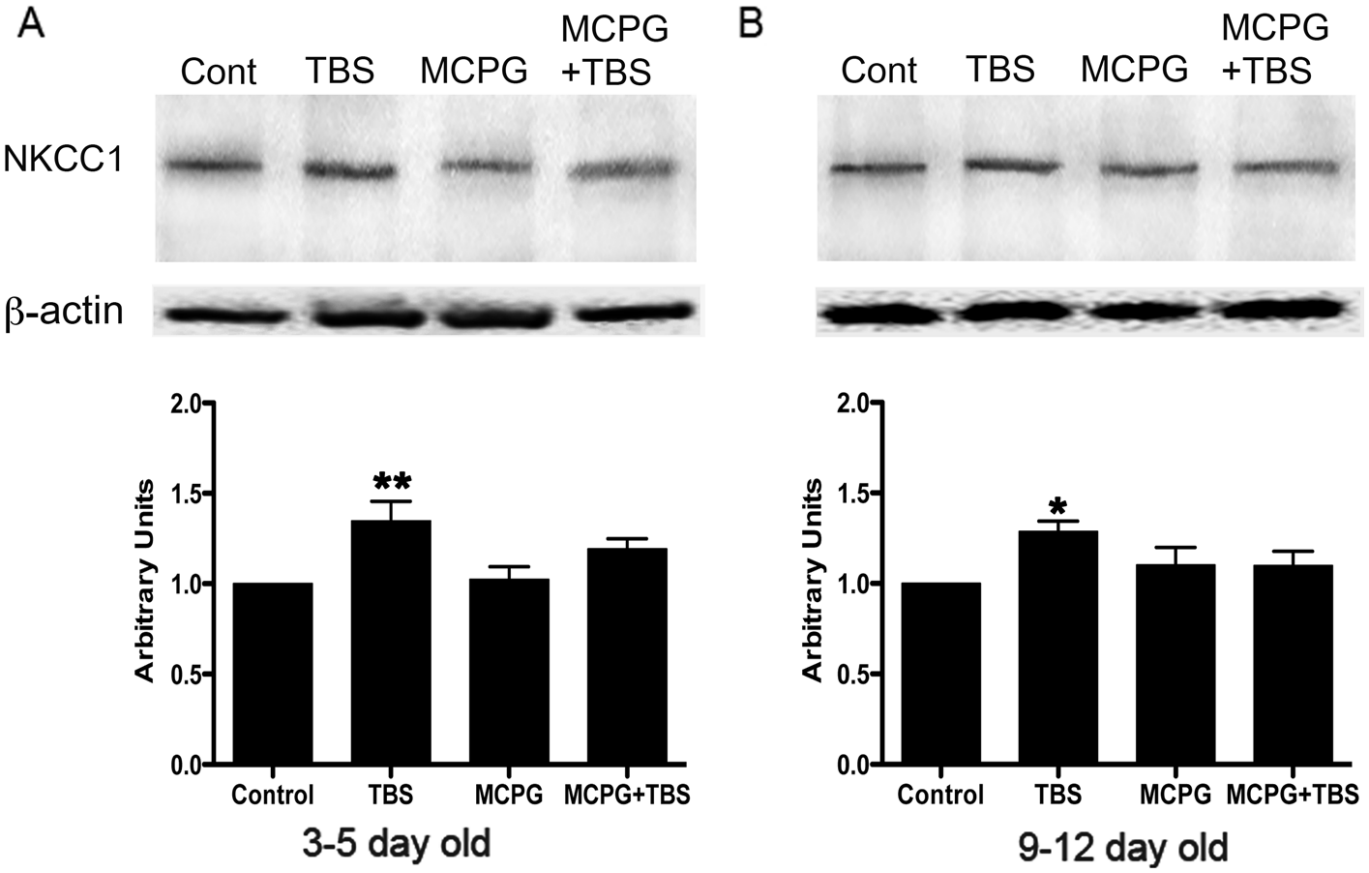
Western blot analysis of NKCC1 in 3–5 day and 9–12 day old rat hippocampus. NKCC1 expression was seen at the expected size of 147 kDa. A) Western blot analysis showing the expression of NKCC1 in the CA1 region of hippocampus from control and treated slices from 3–5 day old rat (upper panel). Densitometric analysis reveals increase in NKCC1 expression in TBS treated, whereas, following MCPG treatment, NKCC1expression was comparable to that in control and no significant changes were seen in MCPG + TBS. B) Western blot analysis depicting comparative changes in the CA1 region of hippocampus in 9–12 day old rats, in control and following treatment (upper panel). Note the increased expression of NKCC1 upon TBS treatment whereas no discernable changes were seen upon MCPG and MCPG + TBS treatment when compared to control. β -actin as loading control and densitometric analysis are shown in middle and bottom panel, respectively. Data are presented as mean ± SD (n = 5 for each group), * P<0.05,** P<0.01.

In neonatal hippocampus, western blots show the expression of NKCC1 at the expected size of >147 kDa. Application of TBS resulted in an increase in NKCC1 expression; whereas, NKCC1 immunoreactivity was not changed with MCPG or MCPG+TBS treatment in the 3–5 day old rat. In the 9–12 day old rat, NKCC1 expression, in comparison to 3–5 old rat, was relatively higher in controls and increased in the presence of TBS, whereas, following MCPG or MCPG + TBS, the expression level of NKCC1 was comparable to that in controls (Fig 7).

## Discussion

### Shifts in E_GABA_ affect GABAergic inhibition in hippocampal CA1 pyramidal neurons

The balance between inhibition and excitation is crucially important for the integrative function of neural circuits in the CNS. The regulation of intracellular Cl^-^ homeostasis plays a critical role in the maintenance of inhibitory neuronal circuit in the CNS [32]. In central neurons, the settlement of steady-state Cl^-^ equilibrium is aided by the co-expression of NKCC1 and KCC2 [33,34]. KCC2 is a principal molecule for the maintenance of low [Cl^-^]_i_ in central neurons [6]. A developmental up-regulation of KCC2 leads to a negative shift in E_Cl_^-^, rendering GABA excitatory in neonates but inhibitory in adult neurons [6,18,31,35]. In line with this thinking, a gradual increase in KCC2 expression was observed in rat hippocampus after birth by western and northern blot analyses [6,24].

In pyramidal cells, changes in [Cl^-^]_i_ via modulation of the activities of co-transporters KCC2 and NKCC1 can lead to changes in E_GABA_ and, hence, GABA-ergic modulation of networks. However, the mechanisms underlying the shifts in E_GABA_ have not been extensively examined until recent years [1,2,3,28,36]. Since imbalances between inhibition and excitation can lead to serious changes in the network behaviour in the CNS, understanding the mechanisms involved in shifts in E_GABA_ is necessary. In the current study, we have investigated the mechanisms underlying TBS-induced shifts in E_GABA_ in rat hippocampal CA1 neurons.

### mGluRs are involved in TBS-induced shift in E_GABA_ in juvenile rat hippocampus via regulation of KCC2 activity

Modulation of KCC2 activity through different mechanisms has been reported in the literature: activity-dependent regulation [37], activation of GABA_A_ receptors [38], activation of tyrosine receptor kinase B (TrkB) receptors [39], postsynaptic Ca^2+^ [36] and protein phosphorylation [40,41] /dephosphorylation [42]. All have been put forward as factors contributing to the regulation of KCC2 activity. However, the exact mechanism underlying the regulation of KCC2 in central neurons is still unclear.

Glutamatergic activity can modulate GABAergic transmission by modifying presynaptic GABA release [43,44,45]. Whether activation of mGluRs is involved in the modulation of E_GABA_ in hippocampal CA1 neurons is unknown. However, there are several lines of evidence suggesting that an investigation of mGluRs as a candidate for regulating KCC2 activity in hippocampal neurons, is worthwhile: a) immunohistochemical studies demonstrate that both group I mGluRs [11] and KCC2 [16] are expressed in somatic and dendritic membranes of hippocampus pyramidal neurons; b) activation of mGluRs (especially group I mGluRs) can lead to an up-regualtion of KCC2 [15](Banke and Gegelashvili, 2008) and NKCC1 [20,21,46] in cortical neurons; c) KCC2 function is regulated by intracellular Ca^2+^ via a PKC-dependent phosphorylation in hippocampal cultured cells [36]; d) activation of group I mGluRs leads to an increase in [Ca^2+^]_i_ via VGCCs [21].

In the present study, our data indicate that mGluRs are involved in TBS-induced shifts in E_GABA_ via modulation of KCC2 activity. MCPG treatment did not significantly change the expression of KCC2 in both juvenile and neonatal hippocampal CA1 neurons. However, it has been recently reported that mGluRs tonically regulate KCC2 levels in the CA3 region [15]. If a similar tonic control exists at the interneuron level in the CA1 region, one can expect a reduction in overall KCC2 activity with mGluR blockade while the control E_GABA_ in pyramidal neurons is unaffected. Our results suggest that this “tonic control mechanism” may not exist in the CA1 region. Further investigation is necessary to confirm this line of thinking.

### mGluRs are also involved in TBS-induced shift in E_GABA_ in neonatal rat hippocampus via modulation of NKCC1 activity

NKCC1 accounts for the regulation of E_GABA_ in neonatal neurons via an accumulation of Cl^-^ in neurons. Under physiological conditions, application of bumetanide can lead to a negative shift in E_GABA_ in rat cortical neurons [47]. Moreover, NKCC1 knockout mice have a more negative E_GABA_ than that in wild-type neurons [48]. Synergistic excitatory action of GABA_A_ and glutamatergic receptors has already been suggested in the neonatal hippocampus [49]. Since group I mGluRs are expressed in hippocampal neurons in the early developmental stage [11,50], whether mGluRs contribute to the regulation of GABA excitatory response via changes in NKCC1 activity and E_GABA_ in neonatal neurons is worth examining. It has been reported that activation of group I mGluRs-mediated signal-transduction pathways can stimulate NKCC1 activity in neonatal cortical neurons [21]. In the present study, our data suggest that mGluRs are involved in TBS-induced positive shift in E_GABA_ via the regulation of NKCC1 activity in neonatal hippocampal neurons.

### mGluRs subtypes involved in TBS-induced shifts in E_GABA_

In juveniles, MCPG blocks the induction of the negative shift in E_GABA_ by TBS, indicating the involvement of mGluRs. Since EGLU did not interfere with the induction of the shift, it is unlikely that group II mGluRs are involved. Among group I mGluRs, MCPG blocks both mGluR1 and mGluR5. MPEP, which blocks mGluR5, but not mGluR1, did not block the TBS-induced shift in E_GABA_, suggesting that mGluR1 is involved in the process. Similarly, LY367385, which blocks mGluR1, but not mGluR5, also did not block the TBS-induced shift in E_GABA_, suggesting that mGluR5 is involved in the process. Therefore, it appears that activation of either mGluR1 or mGluR5, individually, can cause the TBS-induced shift in E_GABA_.

In neonates, MCPG, but not EGLU, blocks the induction of the positive shift in E_GABA_ by TBS, indicating the involvement of group I mGluRs. Application of either LY367385 or MPEP alone is able to block the TBS-induced positive shift in E_GABA_, suggesting that both mGluR1 and mGluR5 are involved in the process. Therefore, in neonates, activation of either mGluR1 or mGluR5 is insufficient to cause the TBS-induced positive shift in E_GABA_. A co-activation of both receptor sub-types appears to be necessary.

The difference between neonates and juveniles in the involvement of mGluR1 and mGluR5 is intriguing. In the neonate, the extra-synaptic localization of mGluR1 and mGluR5 in both pyramidal cells and interneurons [11], perhaps, can facilitate the synergistic activation of these two receptors. In addition, the cooperativity may occur somewhere between downstream elements (such as G-proteins and second messengers) of their respective signal transduction systems. Nevertheless, this synergistic effect of mGluR1 and mGluR5 is not necessary in juvenile hippocampal neurons as either activation of mGluR1 or mGluR5 alone is capable of inducing a negative shift in E_GABA_. Further investigation is necessary to explore mechanisms involved in the above-mentioned age-related differences in mGluR involvement. In addition, future experiments involving the actions of selective agonists at mGluR1 and mGluR5 receptors should be carried out to strengthen the conclusions using antagonists.

### Significance of the dual modulation of mGluRs on KCC2 or NKCC1 activity in hippocampal neurons

mGluRs seem to be involved in differentially modulating the TBS-induced shifts in juvenile and neonatal hippocampal neurons via regulation of KCC2 or NKCC1 activity, respectively, reinforcing the excitatory role of GABA in neonatal neurons while helping in establishing its role as an inhibitory transmitter in juveniles. If mGluRs are involved in regulating KCC2 and NKCC1, these effects will have serious consequences for network behaviour, since GABA-ergic IPSCs significantly contribute to their modulation. In addition, if the actions are different in neonates and juveniles, that adds another dimension to the modulation. Therefore, therapeutic strategies will benefit from taking the new findings into account while developing drugs to treat CNS disorders, involving GABA, in different age groups.

## Conclusions

Our results indicate that changes in the activity of KCC2 or NKCC1 are responsible for TBS-induced shifts in E_GABA_ in juvenile and neonatal rat hippocampal CA1 neurons, respectively. mGluRs seem to be involved in the regulation of KCC2 or NKCC1 in rat hippocampus.
