# Correction: Regulation of GABA Equilibrium Potential by mGluRs in Rat Hippocampal CA1 Neurons

**DOI:** 10.1371/journal.pone.0143266

**Published:** 2015-11-12

**Authors:** Bo Yang, Padmesh S. Rajput, Ujendra Kumar, Bhagavatula R. Sastry

The following information is missing from the Funding section: This study was also supported by the Natural Sciences and Engineering Research Council of Canada (NSERC), grant # RGP-402594-11 to BRS and UK.
